# Inhibition of cystathionine-γ-lyase controls interleukin-12 production by dendritic cells, delayed-type hypersensitivity and transplant rejection

**DOI:** 10.1186/1479-5876-10-S3-P62

**Published:** 2012-11-28

**Authors:** Romain Vuillefroy de Silly, Flora Coulon, Nicolas Poirier, Vojislav Jovanovic, Sophie Brouard, Véronique Ferchaud-Roucher, Gilles Blancho, Bernard Vanhove

**Affiliations:** 1 INSERM, UMR 1064, ITUN, Nantes, France; 2 University of Nantes, Nantes University Hospital Center, Nantes, France; 3 INSERM, UMR915, Nantes, France

## Background

γ-cystathionase (CSE) is a rate-limiting enzyme of the trans-sulfuration pathway which converts methionine and cystathionine into cysteine and H_2_S. T cells are deficient in CSE and cysteine import and therefore are metabolically dependent on accessory cells for cysteine supply.

## Methods and results

In the current study, we demonstrated that pharmacological blockade of CSE with the irreversible inhibitor propargylglycine (PPG) delayed heart allograft rejection (median survival of 26.5 days instead of 9 in controls) and abrogated type IV hypersensitivity to keyhole limpet haemocyanin (Th-1 response), but did not modify antibody responses (Th-2 response). The dominant biological effect of CSE blockade was a repression of the IL-12 p40, T-Bet and IL-1β transcripts inside the graft paralleled with a decrease, at the protein level, of IL-12 and IFN-γ. In parallel, we found that tolerance induced by costimulation blockade or immunosuppression after heart and kidney allotransplantation in the LEW.1W to LEW.1A rat model was associated with a two to five fold repression of intragraft CSE, as well as of several other enzymes of the trans-sulfuration pathway. Monocytes and Dendritic cells treated by PPG or by the reversible CSE inhibitor, β-cyano alanine, as well as by siRNA specific for CSE, dose-dependently and differentially regulated production of IL-12 cytokine. The effect was independent of NFkB or H_2_S production, but could be assigned to a modulation of intracellular cysteine content.

## Conclusion

Our results identify CSE as a novel factor that plays a critical role in IL-12 production by monocytes and DCs by modulating intracellular cysteine levels, which in turn controls Th-1 type immune responses.

